# Expanding Bioactive Fragment Space with the Generated Database GDB-13s

**DOI:** 10.1021/acs.jcim.3c01096

**Published:** 2023-09-18

**Authors:** Ye Buehler, Jean-Louis Reymond

**Affiliations:** Department of Chemistry, Biochemistry and Pharmaceutical Sciences, University of Bern, Freiestrasse 3, 3012 Bern, Switzerland

## Abstract

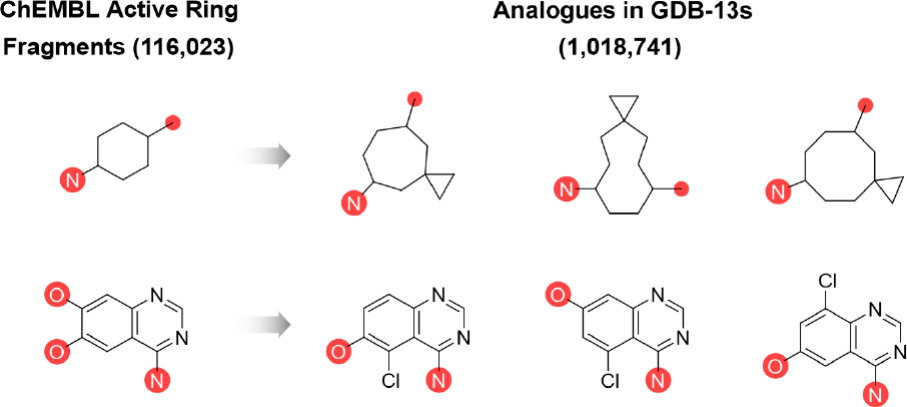

Identifying innovative fragments for drug design can help medicinal chemistry address new targets and overcome the limitations of the classical molecular series. By deconstructing molecules into ring fragments (RFs, consisting of ring atoms plus ring-adjacent atoms) and acyclic fragments (AFs, consisting of only acyclic atoms), we find that public databases of molecules (i.e., ZINC and PubChem) and natural products (i.e., COCONUT) contain mostly RFs and AFs of up to 13 atoms. We also find that many RFs and AFs are enriched in bioactive vs inactive compounds from ChEMBL. We then analyze the generated database GDB-13s, which enumerates 99 million possible molecules of up to 13 atoms, for RFs and AFs resembling ChEMBL bioactive RFs and AFs. This analysis reveals a large number of novel RFs and AFs that are structurally simple, have favorable synthetic accessibility scores, and represent opportunities for synthetic chemistry to contribute to drug innovation in the context of fragment-based drug discovery.

## Introduction

Medicinal chemistry becomes an increasingly retrospective activity as public databases such as PubChem^1^ and ChEMBL^2^ list increasing numbers of known drug-like molecules and their biological activity, from which new analogues can be derived. Nevertheless, introducing chemical novelty in new drugs is important because it can help to address new target types and overcome the limitations of classical molecular series in terms of physicochemical properties, selectivity, toxicity, and metabolism, as well as to secure intellectual property and the possibility of commercial development.^3−6^ Currently, innovation focuses on exploiting very large libraries of screening compounds obtained by combining known building blocks using known chemistry.^7,8^ These libraries contain billions of molecules, as in ZINC^9^ or the Enamine REAL database,^10,11^ up to hundreds of billions of molecules in DNA encoded libraries,^12−15^ or even much larger numbers of peptides and cyclic peptides in phage or ribosome display libraries.^16,17^ Such molecules often break Lipinski’s rule of five but can nevertheless be developed as drugs.^18,19^

Despite the impressive numbers of molecules in the above-mentioned databases, these molecules are obtained by combining a limited set of building blocks, typically up to thousands (only 20 for genetically encoded peptides), which severely limits fragment diversity. With respect to fragments, an additional, potentially more important, but mostly unexploited reservoir of novelty exists in the generated databases (GDBs), which systematically enumerate molecules of up to 11, 13, or 17 non-hydrogen atoms (heavy atom count (HAC) = 11, 13, or 17) from mathematical graphs using simple rules of chemical stability and synthetic feasibility.^20−23^ For instance, the GDBs feature molecules with many unprecedented molecular frameworks (graphs including rings and linker bonds).^24,25^

**Figure 1:**
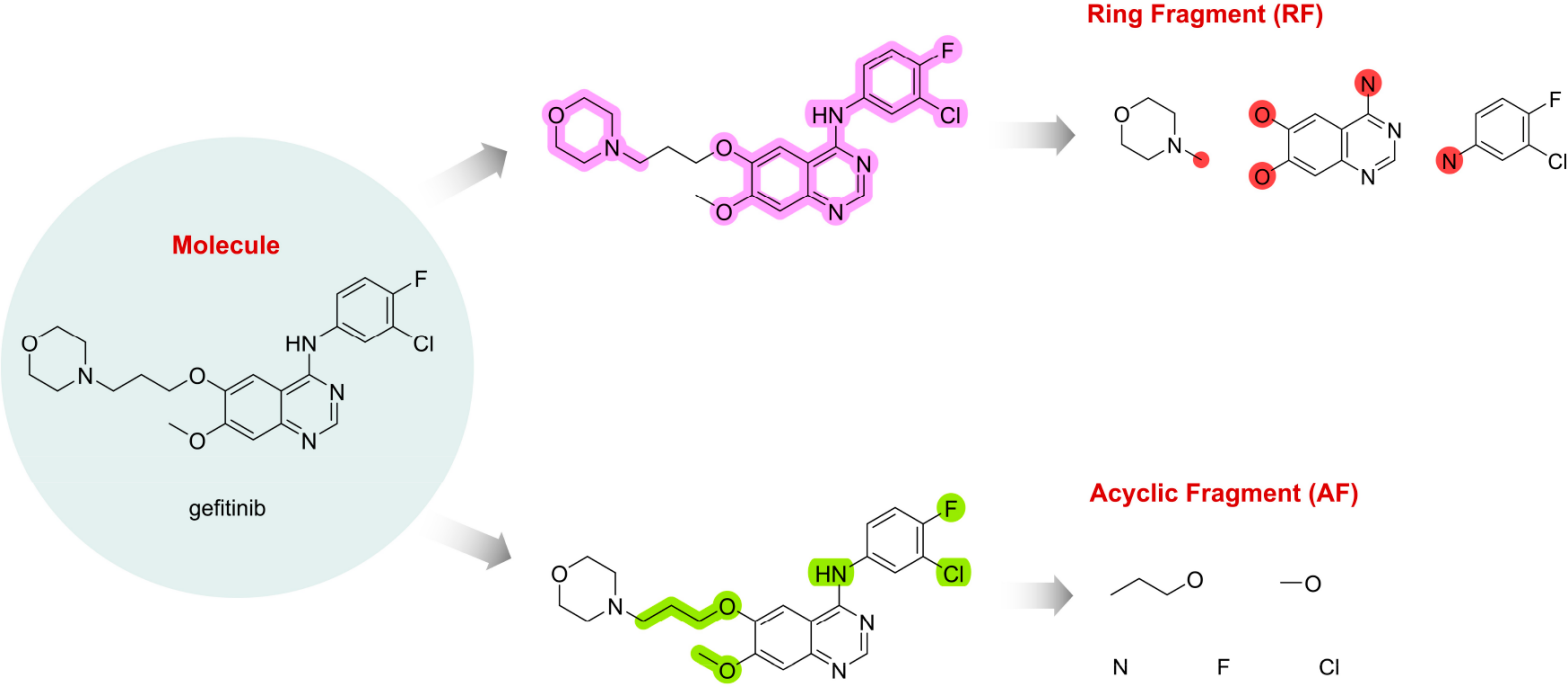
Fragmentation of molecules into ring fragments (RFs) and acyclic fragments (AFs). The general principle is given in the example of the drug gefitinib. For RFs, acyclic atoms are labeled in red.

Here, we propose an approach to identify novel fragments from the GDBs that could be useful for drug design by taking the accumulated knowledge of bioactive compounds into account through an analysis of fragments. First, we assess the known chemical space by deconstructing molecules in the public databases ZINC (screening compounds),^9^ PubChem (published molecules),^1^ and COCONUT (natural products and NP-like molecules)^26^ into ring fragments (RFs, obtained by removing all atoms not directly connected to a ring) and acyclic fragments (AFs, obtained by removing all ring atoms) (Figure 1). This fragmentation is inspired by computational retrosynthetic analyses such as RECAP,^27^ rdScaffoldNetwork,^28^ DAIM,^29^ BRICS,^30^ CCQ,^31^ eMolFrag,^32^ molBLOCKS,^33^ or Fragmenter.^34^ In the present context, our deconstruction into RFs and AFs is designed to simplify molecules and focus on structural types. Interestingly, most molecules in ZINC, PubChem, and COCONUT break down into RFs and AFs of 13 atoms or less.

In the second part of our approach, we identify RFs and AFs which are strongly enriched in bioactivity compared to inactive molecules in ChEMBL (target annotated compounds)^2^ and search for analogues of these fragments in RFs and AFs derived from the generated database GDB-13s.^25^ This database is a 10% subset of the database GDB-13,^20^ which lists 970 million small molecules of up to 13 atoms exhaustively enumerated from mathematical graphs following the simple rules of chemical stability and synthetic feasibility. While GDB-13 excludes strained rings (e.g., cubane and prismane) and hydrolytically labile and reactive functional groups (e.g., hemiacetals, aminals, enols, acyl chlorides, isocyanides, peroxides, azides, and thiols) and only considers C, N, O, S, and Cl as elements, GDB-13s additionally excludes non-aromatic olefins, acetals, enol ethers, aziridines, and aldehydes, which only rarely occur in drug molecules. Nevertheless, GDB-13s contains many unprecedented molecular frameworks (graphs including rings and linker bonds).^24,25^ In the present analysis, we find that many of the bioactive-like RFs and AFs identified in GDB-13s are structurally relatively simple and have favorable synthetic accessibility scores (SAscores)^35^ and therefore represent opportunities for synthetic chemistry to contribute to drug innovation in the context of fragment-based drug discovery.^36,37^

## Results and Discussion

### Fragment Analysis of Known Molecules and GDB-13s

**Figure 2:**
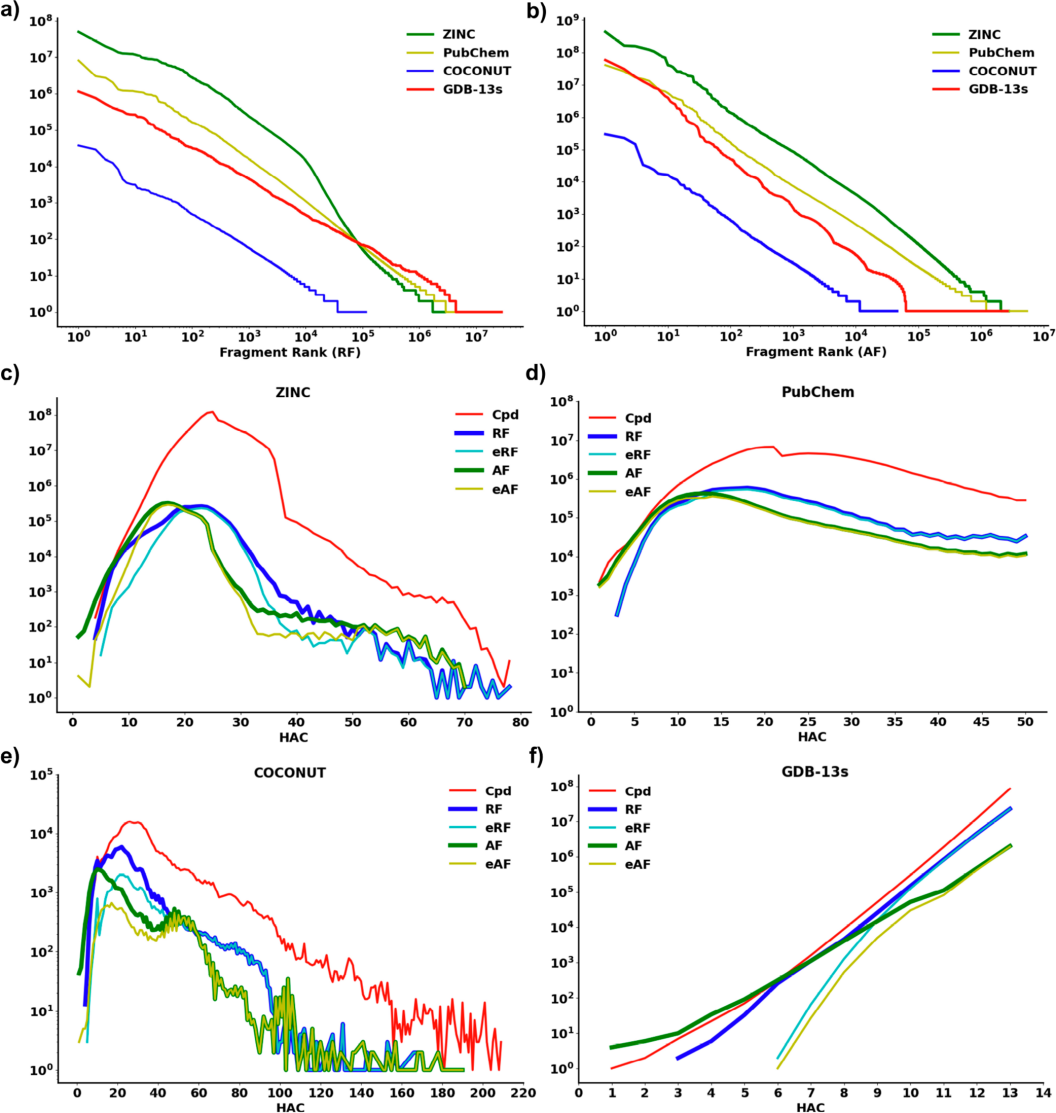
Frequency distribution of (a) ring fragments (RFs) and (b) acyclic fragments (AFs) in ZINC, PubChem, COCONUT, and GDB-13s. Count of compounds (Cpds), RFs, exclusive ring fragments (eRFs), AFs, and exclusive acyclic fragments (eAFs) in (c) ZINC, (d) PubChem, (e) COCONUT, and (f) GDB-13s as a function of the heavy atom count (HAC). The curves of RF and AF are depicted thicker than the other curves to help visualize the distribution in the regions with a high overlap.

To assess the known chemical space, we extracted RFs and AFs from 885 905 524 molecules in the ZINC database,^9^ 100 852 694 molecules of up to 50 non-hydrogen atoms in PubChem,^1^ and 401 624 natural products (NPs) and NP-like molecules in COCONUT.^26^ We also extracted RFs and AFs from the 99 394 177 molecules in GDB-13s,^25^ to be used as a source of novelty later in the study. In all these databases, the number of molecules per RF and AF followed a typical power law distribution, with few RFs and AFs occurring in many molecules and a relatively large number of RFs and AFs occurring only once, referred to as singletons (Figures 2a and 2b and Table 1). The most frequent RFs and AFs in each database were rather small, featuring mono- and disubstituted benzene rings and azacycles for RFs in known molecules, cyclopropanes for RFs in GDB-13s, and single-atom groups for AFs in all databases (Figures S1 and S2). In fact, although the size distribution of the compounds, RFs, and AFs in known molecules extended far beyond 13 atoms (Figures 2c–2f), the RFs and AFs up to 13 atoms were sufficient to cover most molecules except for the natural products in COCONUT, which feature many molecules with RFs larger than 13 atoms (Table 1, entry numbers 2–4). While fragments shared by the four databases were often structurally simple, those occurring in only one of the four databases analyzed (exclusive fragments, eRF and eAF) were generally more complex, as exemplified by the most frequent cases (Figures S3 and S4).

**Table 1:** Molecule and Fragment Counts in Different Databases

no.^a^		ZINC		PubChem		COCONUT		GDB-13s	
1	cpds^b^	885 905 524		100 852 694		401 624		99 394 177	
2	cpds from RF ≤ 13^c^	743 430 899	83.9%	68 876 892	68.3%	132 432	33.0%	99 394 177	100%
3	cpds from AF ≤ 13^d^	818 548 834	92.4%	94 526 506	93.7%	357 976	89.1%	99 394 177	100%
4	cpds from ARF ≤ 13^e^	678 518 591	76.6%	62 998 179	62.5%	98 990	24.6%	99 394 177	100%
5	RF	2 838 201		9 037 484		115 381		28 246 012	
6	eRF^f^	2 165 176	76.3%	8 139 719	90.1%	45 448	39.4%	28 011 035	99.2%
7	RF, singleton^g^	1 115 630	39.3%	6 111 177	67.6%	78 920	68.4%	23 842 697	84.4%
8	RF ≤ 13^h^	158 576	5.6%	1 746 923	19.3%	17 211	14.9%	28 246 012	100%
9	eRF ≤ 13^i^	17 578	0.6%	1 333 179	14.8%	1863	1.6%	28 011 035	99.2%
10	RF ≤ 13, singleton^j^	58 749	2.1%	1 048 461	11.6%	10 244	8.9%	23 842 697	84.4%
11	AF	2 756 691		5 466 187		45 816		2 640 023	
12	eAF^f^	2 319 553	84.1%	4 722 488	86.4%	18 608	40.6%	2 447 627	92.7%
13	AF, singleton^g^	688 408	25.0%	4 256 810	77.9%	34 243	74.7%	2 576 927	97.6%
14	AF ≤ 13^h^	338 990	12.3%	2 225 960	40.7%	17 216	37.6%	2 640 023	100%
15	eAF ≤ 13^i^	145 340	5.3%	1 805 294	33.0%	2131	4.7%	2 447 627	92.7%
16	AF ≤ 13, singleton^j^	52 606	1.9%	1 535 039	28.1%	9950	21.7%	2 576 927	97.6%

a no. = entry number.

b cpds = compounds/molecules.

c cpds from RF ≤ 13 = molecules covered by ring fragments (RFs) with a heavy atom count (HAC) of up to 13.

d cpds from AF ≤ 13 = molecules covered by acyclic fragments (AFs) with an HAC of up to 13.

e cpds from ARF ≤ 13 = molecules covered by both RFs and AFs with an HAC of up to 13.

f eRF/eAF = exclusive RF/AF, absent from the other three databases.

g RF/AF, singleton = RF/AF with only a single molecule example.

h RF ≤ 13/AF ≤ 13 = RFs/AFs with an HAC of up to 13.

i eRF ≤ 13/eAR ≤ 13 = exclusive RFs/AFs with an HAC of up to 13, absent from the other three databases.

j RF ≤ 13, singleton/AR ≤ 13, singleton = RF ≤ 13/AF ≤ 13 with only a single molecule example. RF and AF subcategories are calculated relative to total RFs and AFs, respectively.

Within the space covered by RFs and AFs of up to 13 atoms, GDB-13s largely outnumbered the known molecules in terms of RFs, resulting in a high percentage of exclusive RFs (99.2% eRFs ≤ 13 atoms, Table 1, entry number 9). Most AFs ≤ 13 atoms in GDB-13s were also exclusive (92.7% eAFs ≤ 13 atoms, Table 1, entry number 15), although the absolute number of AFs in GDB-13s was comparable to the number of AFs in ZINC and smaller than the number of AFs in PubChem. In fact, PubChem, ZINC, and COCONUT also contained many exclusive eRFs ≤ 13 atoms and eAFs ≤ 13 atoms, reflecting that the enumeration of GDB-13s excluded strained rings and certain functional groups and only considered C, N, O, S, and Cl as elements. Nevertheless, the above analysis showed that GDB-13s contained a very large number of both eRFs and eAFs and could therefore serve as a source of novel RFs and AFs to expand the space of known molecules.

### Comparative Analysis of RFs and AFs in ChEMBL Active and Inactive Molecules

Aiming to select novel fragments in GDB-13s by exploiting knowledge on bioactive compounds, we analyzed molecules from the ChEMBL database to test if different RFs and AFs were associated with active or inactive compounds.^2^ We selected the 2 136 218 ChEMBL molecules with an HAC ≤ 50, separated them into 560 230 actives (IC_50_ or EC_50_ ≤ 10 μM, ChEMBL_actives) and 1 575 988 inactives (all others, ChEMBL_inactives), and extracted the corresponding RFs and AFs. For each RF and AF, we computed its total occurrence as the number of ChEMBL molecules containing this RF or AF, its relative occurrence in active molecules (% active) and inactive molecules (% inactive), and its activity ratio *R*_bioactive_ = (% active)/(% inactive).

**Figure 3:**
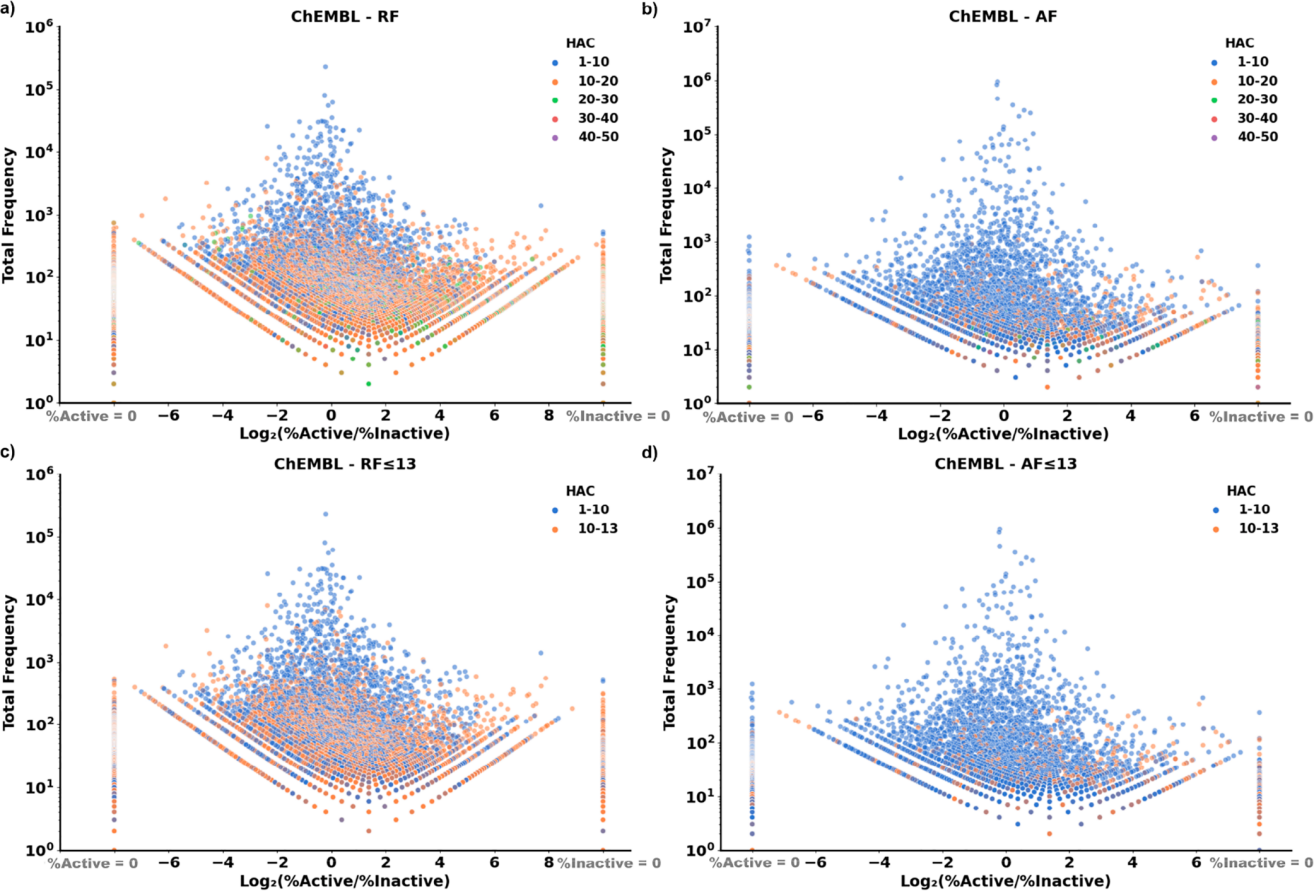
Volcano plots visualizing all active and inactive fragments extracted from ChEMBL. The logarithm value (base 2) of the ratio of the proportion of fragments in all active molecules to the proportions of fragments in all inactive molecules, namely, log_2_(% active/% inactive), was plotted on the *x*-axis, and the total frequency (the sum of the occurrences of the fragments in active molecules and in inactive molecules) was plotted on the *y*-axis. The colors of the data points indicate the heavy atom count (HAC) range of the fragments. Occurrences of fragments that only appeared in inactive compounds (% active = 0) were displayed vertically in a straight line at the left end of the plot, while occurrences of fragments that only appeared in active compounds (% inactive = 0) were displayed vertically in a straight line at the right end of the plot.

A volcano scatter plot of the total occurrence of each RF or AF as a function of *R*_bioactive_ showed that RFs and AFs spanned a broad range of *R*_bioactive_ values and total occurrences (Figures 3a and 3b). The situation was similar when only fragments of up to 13 atoms were analyzed (Figures 3c and 3d). From this analysis, we partitioned ChEMBL fragments according to their *R*_bioactive_ values into active (*R*_bioactive_ ≥ 4), inactive (*R*_bioactive_ ≤ 0.25), or nonpreferential fragments (intermediate values, *R*_bioactive_ ≈ 1). While the most frequent fragments were small and nonpreferential, many fragments, including all singletons, occurred exclusively in either the ChEMBL_actives or ChEMBL_inactives subset and were accordingly assigned to either the active (*R*_bioactive_ ≥ 4) or inactive (*R*_bioactive_ ≤ 0.25) subset, respectively (Table 2). The top 10 most frequent active (*R*_bioactive_ ≥ 4) and inactive (*R*_bioactive_ ≤ 0.25) RFs and AFs in ChEMBL were all in the size range of GDB-13s. Four of these top 10 active RFs featured halogenated benzene rings, while four of the top 10 inactive RFs were saturated heterocycles (Figure S5). For AFs, fluorine prevailed in four of the top 10 active AFs, while sulfur occurred in four of the top 10 inactive AFs (Figure S6).

**Table 2:** RF/AF Analysis of the ChEMBL_actives and ChEMBL_inactives Subsets

no.^a^		ChEMBL_actives		ChEMBL_inactives		*R*_bioactive_ ≥ 4		*R*_bioactive_ ≈ 1		*R*_bioactive_ ≤ 0.25	
1	cpds^b^	543 971		1 575 988							
2	cpds from RF ≤ 13^c^	215 243	39.6%	870 442	55.2%						
3	cpds from AF ≤ 13^d^	523 674	96.3%	1 509 677	95.8%						
4	cpds from ARF ≤ 13^e^	198 367	36.5%	813 618	51.6%						
5	RF	145 174		300 613		116 023		25 197		266 255	
6	eRF^f^	106 862	73.6%	262 301	87.3%	106 862	92.1%	0	0%	262 301	98.5%
7	RF, singleton^g^	93 023	64.1%	193 248	64.3%	78 758	67.9%	0	0%	182 620	68.6%
8	RF ≤ 13^h^	28 309	19.5%	55 143	18.3%	15 211	13.1%	10 883	43.2%	40 930	15.4%
9	eRF ≤ 13^i^	11 881	8.2%	38 715	12.9%	11 881	10.2%	0	0%	38 715	14.5%
10	RF ≤ 13, singleton^j^	12 260	8.5%	23 463	7.8%	7642	6.6%	0	0%	20 699	7.8%
11	AF	26 482	4.7%	81 690	5.2%	16 567		8605		71 125	
12	eAF^f^	14 613	55.2%	69 817	85.5%	14 613	88.2%	0	0%	69 817	98.2%
13	AF, singleton^g^	15 773	59.6%	49 745	60.9%	11 252	67.9%	0	0%	46 974	66.0%
14	AF ≤ 13^h^	16 137	60.9%	45 091	55.2%	7875	47.5%	7063	82.1%	36 498	51.3%
15	eAF ≤ 13^i^	6347	24.0%	35 301	43.2%	6347	38.3%	0	0%	35 301	49.6%
16	AF ≤ 13, singleton^j^	8008	30.2%	22 540	27.6%	4638	28.0%	0	0%	20 689	29.1%

a no. = entry number.

b cpds = compounds/molecules.

c cpds from RF ≤ 13 = molecules covered by ring fragments (RFs) with a heavy atom count (HAC) of up to 13.

d cpds from AF ≤ 13 = molecules covered by acyclic fragments (AFs) with an HAC of up to 13.

e cpds from ARF ≤ 13 = molecules covered by both RFs and AFs with an HAC of up to 13.

f eRF/eAF = exclusive RF/AF, absent from the other three databases.

g RF/AF, singleton = RF/AF with only a single molecule example.

h RF ≤ 13/AF ≤ 13 = RF/AF with an HAC of up to 13.

i eRF ≤ 13/eAR ≤ 13 = exclusive RFs/AFs with an HAC of up to 13, absent from the other three databases.

j RF ≤ 13, singleton/AR ≤ 13, singleton = RF ≤ 13/AF ≤ 13 with only a single molecule example. The RF and AF subcategories are calculated relative to total RFs and AFs, respectively.

**Figure 4:**
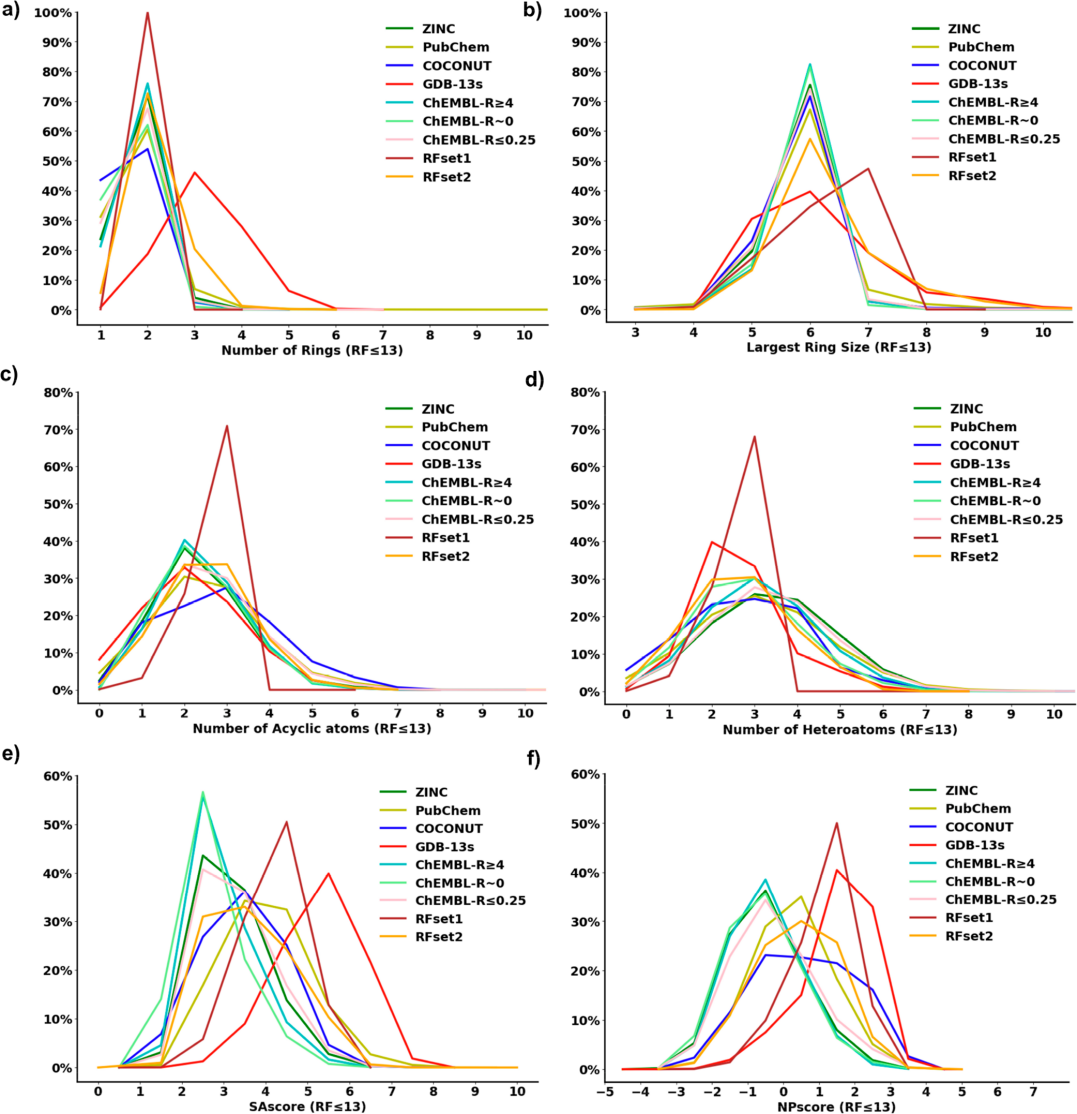
Frequency histograms of ring fragments (RFs) from the various databases and subsets for (a) the number of rings, (b) the largest ring size, (c) the number of acyclic atoms, (d) the number of heteroatoms, (e) the synthetic accessibility score (SAscore), and (f) the natural product likeness score (NPscore).

While many RFs and AFs occurred preferentially in either the ChEMBL_active or ChEMBL_inactive molecules, these fragments did not differ strongly from each other or from RFs and AFs in known molecules (PubChem, ZINC, and COCONUT) in terms of overall structural features. Indeed, the different data sets of known molecules had quite similar property profiles for RFs of up to 13 atoms in terms of the number of rings, the largest ring size, and the number of acyclic atoms and heteroatoms (Figures 4a–4d). Similarly, AFs of up to 13 atoms in these data sets had comparable property profiles concerning the number of quaternary centers, triple bonds, heteroatoms, and terminal atoms (Figures S7a–S7d).

On the other hand, the property profiles of GDB-13s RFs and AFs were clearly different from those of known molecules. For instance, RFs from GDB-13s had a broader distribution in terms of the number of rings and the largest ring size and fewer heteroatoms than the different RF data sets of known molecules. Furthermore, the GDB-13s AFs stood out with a larger number of triple bonds and terminal atoms compared to the AF data sets of known molecules. These differences probably explained the less favorable synthetic accessibility score (SAscore) of the GDB-13s RFs and AFs (Figures 4e and S7e).^35^ Indeed, the SAscore is based on the presence of substructures frequently found in known molecules. Note that the GDB-13s RFs and AFs had relatively high natural product likeness scores (NPscores),^38^ comparable to those of the COCONUT molecules (Figures 4f and S7f). The high NPscores of the GDB-13s RFs and AFs probably reflect the high percentage of non-aromatic, stereochemically complex structures in GDB-13s since the NPscore assigns higher values for the presence of such structural features.

### Bioactivity-Guided Selection of RFs and AFs in GDB-13s

The analysis presented above suggested two possible approaches to select RFs and AFs from GDB-13s for drug design. First, the narrower structural parameter ranges covered by RFs and AFs from known molecules, active or inactive, which correlated with their more favorable SAscores compared to the GDB-13s RFs and AFs, indicated to select GDB-13s fragments with limited structural complexity, which would certainly help with a possible synthesis. Following up on this idea, we selected a subset of GDB-13s RFs and AFs by constraining the structural parameters closer to known molecules but considering only those exclusive to GDB-13s to ensure novelty. To our delight, this selection resulted in a sizable number of GDB-13s fragments. Indeed, we obtained 960 587 GDB-13s eRFs with up to two rings, a ring size up to seven, up to three heteroatoms, and three acyclic atoms, named RFset1. For the selection of AFs from GDB-13s, we obtained 462 439 GDB-13s eAFs without any quaternary center and up to one triple bond, up to four heteroatoms, and up to four terminal atoms, named AFset1.

In a second, narrower selection, we assumed that ChEMBL-derived RFs and AFs in the *R*_bioactive_ ≥ 4 value range (defined as active fragments) reflected privileged structural types, while those in the *R*_bioactive_ ≤ 0.25 value range (defined as inactive fragments) marked undesirable structural types in terms of possible bioactivities. To expand the scope of the ChEMBL active fragments, we retrieved all GDB-13s RFs and AFs within a Jaccard distance *d*_J_ ≤ 0.6 of any of the ChEMBL active fragments, using the MAP4 fingerprint as a similarity measure.^39^ In this manner, we obtained 97 664 RFs and 43 704 AFs, from which we removed the 25 162 RFs and 15 484 AFs found within *d*_J_ ≤ 0.6 of any inactive fragments, leaving 72 502 RFs, named RFset2, and 28 220 AFs, named AFset2, as bioactive-like fragments from GDB-13s. In these sets, many fragments were also exclusive to GDB-13s, ensuring novelty (51 303 eRFs, 70.8%; 17 620 eAFs, 62.4%).

The property profiles of RFset1 and AFset1, which both resulted from constraining structural parameters, remained substantially different from those of known molecules because the frequency peaked at the highest parameter value selected. This distribution reflects the combinatorial enumeration used to generate GDB-13s, which provides many more possible molecules at the largest values of structural parameters. Therefore, the SAscore remained less favorable and the NPscore relatively high in both sets. On the other hand, the property profiles of RFset2 and AFset2, selected by substructure similarity to ChEMBL bioactive fragments, were like those of known molecules, reflecting the structural similarity selection used to compose these sets (Figures 4a–4d and S7a–S7d). RFset2 and AFset2 also displayed lower SAscore and NPscore values than the full sets of GDB-13s RFs and AFs, indicating that they were generally less complex and closer to the RFs and AFs from known molecules (Figures 4e, 4f, S7e, and S7f).

**Figure 5:**
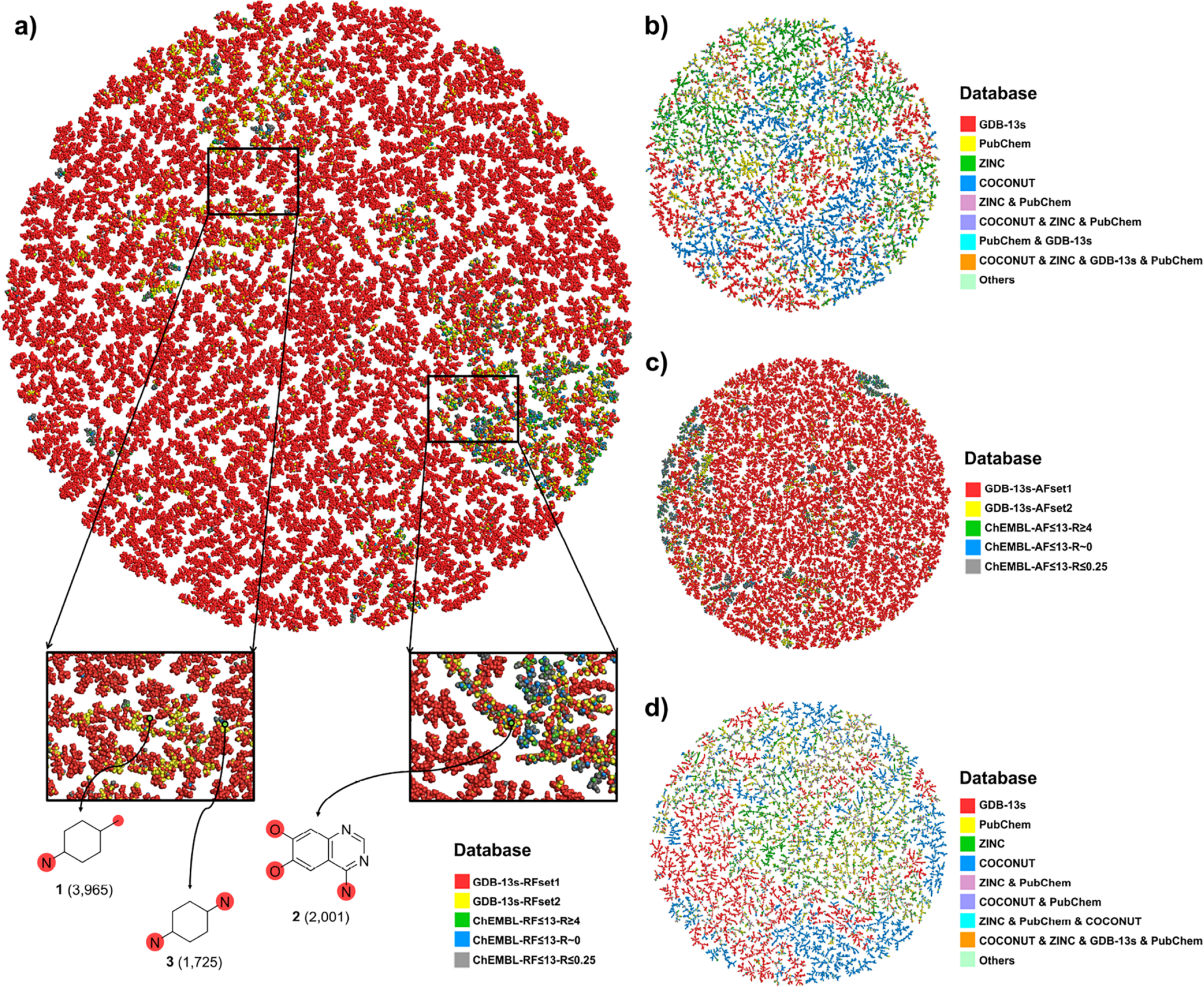
Tree map (TMAP) visualization of (a) the 1 042 610 ring fragments (RFs) from RFset1, RFset2, and ChEMBL; (b) the top 10 000 RFs in ZINC, PubChem, COCONUT, and GDB-13s; (c) the 533 153 acyclic fragments (AFs) from AFset1, AFset2, and ChEMBL; and (d) the top 10 000 AFs in ZINC, PubChem, COCONUT, and GDB-13s, color-coded by the source data sets, the synthetic accessibility score (SAscore), and different properties. An interactive version of the TMAPs is accessible at https://tm.gdb.tools/map4 (MAP4_fused_GDB-13s_RFset1_RFset2_and_ChEMBL; MAP4_4databases_top10k_RF; MAP4_fused_GDB-13s_AFset1_AFset2_and_ChEMBL; MAP4_4databases_top10k_AF).

**Figure 6:**
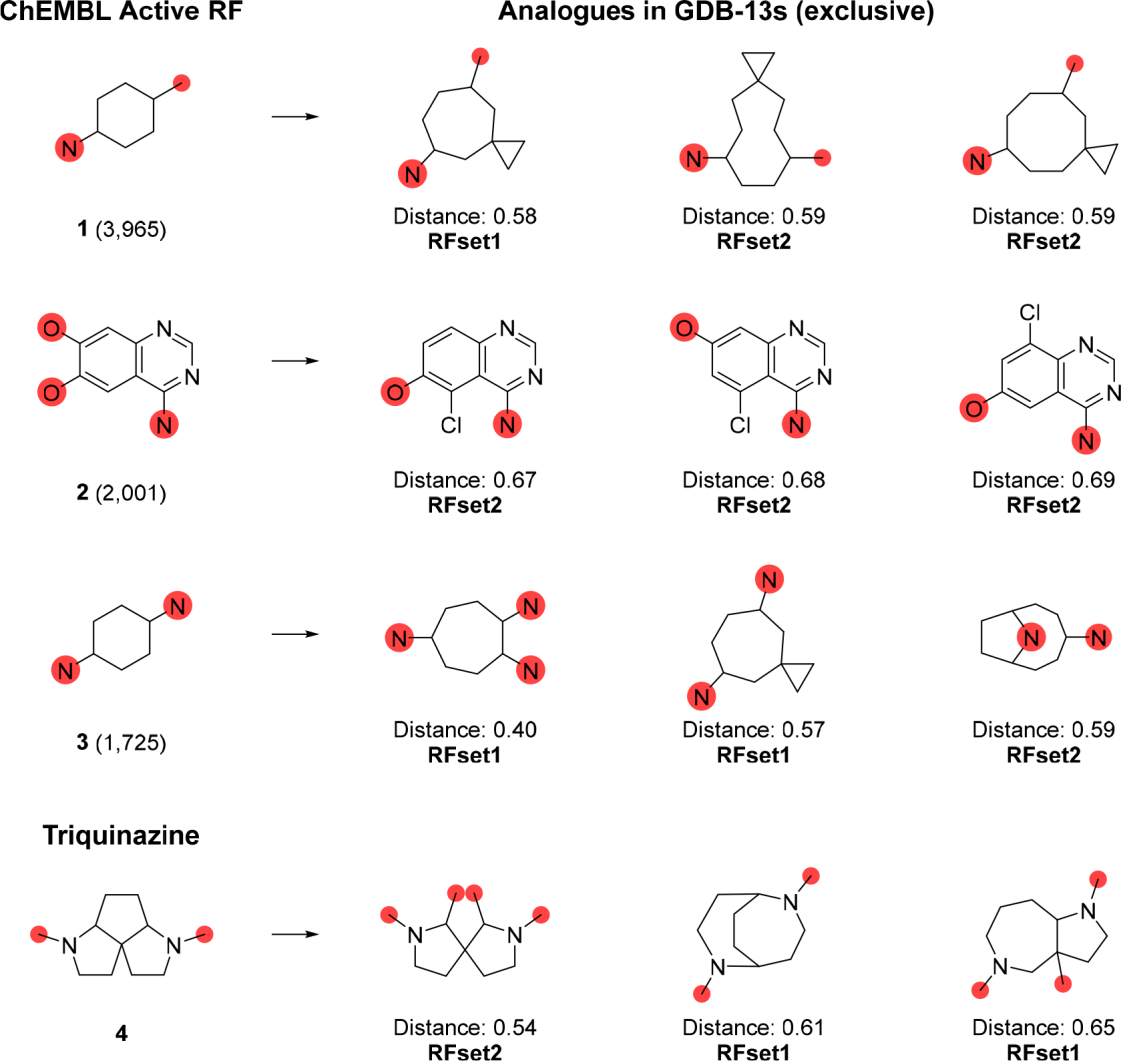
Analogues of highly active ChEMBL ring fragments (RFs) and triquinazine found in the subsets of GDB-13s (RFset1**/**RFset2). The total occurrences of the ChEMBL RFs, or the MAP4 fingerprint jaccard distances between the analogues from GDB-13s and the corresponding ChEMBL Active RF, are indicated below the structures.

To gain a detailed insight into the bioactivity-selected subset of GDB-13s RFs and AFs, we computed interactive TMAPs (tree maps)^40^ using the MinHashed fingerprint MAP4 as a similarity measure (Figure 5).^39^ These interactive TMAPs allow one to browse through the two databases and search for interesting RFs and AFs using various color-coded properties as guides. To illustrate the available options, we searched for novel analogues of the three most frequent active (*R*_bioactive_ ≥ 4) RFs in ChEMBL, one of which occurs in the kinase inhibitor drug gefitinib, revealing potentially interesting analogues (Figure 6). More interesting GDB-13s eRFs are exemplified as analogues of triquinazine, an eRF from GDB-13s previously used as a scaffold for a Janus kinase inhibitor analogue of the known drug tofacitinib.^41^ In principle, the same selection can also be made with the GDB-13s analogues of AFs, as exemplified for the most frequent active (*R*_bioactive_ ≥ 4) AFs from ChEMBL (Figure S8). In this case, however, the selection of interesting AFs is less obvious since the chemistry of AFs highly depends on their connection to RFs.

## Conclusion

In summary, deconstructing known molecules from the ZINC and PubChem databases and natural products from the COCONUT database to form fragments (RFs and AFs) showed that these molecules mostly consist of RFs and AFs of 13 atoms or less. A comparative analysis of the database GDB-13s, which lists 99 million possible molecules of up to 13 atoms, showed that over 99% of the 28 million RFs and 93% of the 2.6 million AFs in GDB-13s are absent from public databases and are therefore exclusive and, in principle, novel. Furthermore, by analyzing the ChEMBL database, we found that certain RFs and AFs occur more frequently in known active vs inactive molecules. Analyzing the properties of active RFs and AFs in ChEMBL to define property and similarity ranges then allowed us to extract one million RFs and half a million AFs from GDB-13s with ChEMBL-active-like features. These ChEMBL-active-like RFs and AFs from GDB-13s are structurally relatively simple and have favorable SAscores and therefore represent attractive targets for synthesizing new fragments with favorable properties for drug design.

## Methods

### Extracting RFs and AFs from Molecules

The RFs and AFs were obtained from molecules by processing their SMILES^42^ using RDkit^43^ as follows (Figure 1). RFs: break all bonds between any two acyclic atoms and remove all acyclic atoms not directly attached to the rings. Acyclic atoms directly connected to more than one ring system are disconnected and reattached to each ring system separately. AFs: break all bonds between the cyclic and acyclic atoms and remove all cyclic atoms.

### TMAPs

Tree maps (TMAPs) were generated by specifying standard parameters^40^ using the MAP4 fingerprint (MinHashed atom-pair fingerprint up to a diameter of four bonds).^39^ MAP4 fingerprints were computed with dimensions of 256.

## Data Availability

GDB-13 (970 million molecules of up to 13 atoms enumerated from graphs under ring strain and functional group restriction criteria, as described earlier)^20^ and GDB-13s (a 99 million molecule subset of GDB-13 with additional functional group restrictions, as described earlier)^25^ are hosted on the open-access repository Zenodo and can be downloaded free of charge at 10.5281/zenodo.7041051. All the molecules are stored in a dearomatized, canonized SMILES format and compressed as a GNU zip archive. The ZINC data used in this study were the February 2022 version (https://zinc.docking.org). The October 2021 version of the PubChem data was first downloaded from the NCBI (National Center for Biotechnology Information), NIH (National Institutes of Health) via an FTP server (https://ftp.ncbi.nlm.nih.gov/pubchem/Compound/CURRENT-Full). Then the compounds with HACs not greater than 50 were extracted to build the PubChem database. The COCONUT data adopted in this study were the February 2021 version (https://github.com/reymond-group/Coconut-TMAP-SVM). ChEMBL_active and ChEMBL_inactive data sets were extracted from ChEMBL31 (https://ftp.ebi.ac.uk/pub/databases/chembl/ChEMBLdb/latest). The Molecule Breakdown Model has been made freely available and is under the MIT license. It was distributed in a GitHub repository upon publication of this manuscript: https://github.com/Ye-Buehler/Molecule_Breakdown_Model.

## Supporting Information Available

Top 10 most populated RFs/AFs in GDB-13s, ZINC, PubChem, and COCONUT; top 20 most frequent RFs shared by the different databases; top 10 eRFs in the different databases; top 10 most frequent RFs and AFs in the active and inactive ChEMBL subsets; frequency histograms of the AFs from the various databases and subsets for the number of quaternary centers, number of triple bonds, number of heteroatoms, number of terminal atoms, SAscore, and NPscore; and analogues of highly active ChEMBL AFs found in GDB-13s AFset1**/**AFset2 (PDF)
