# Supplementary material for: Expanding Bioactive Fragment Space with the Generated Database GDB-13s
Source: J Chem Inf Model. 2023 Sep 18;63(20):6239–48. doi: 10.1021/acs.jcim.3c01096 (PMC10598793; doi:10.1021/acs.jcim.3c01096)
Supplement: Supplementary file 1 — ci3c01096_si_001.pdf [file ci3c01096_si_001.pdf]

Supporting Information for

Expanding Bioactive Fragment Space with the Generated Database GDB-13s

Ye Buehler and Jean-Louis Reymond*

Department of Chemistry, Biochemistry and Pharmaceutical Sciences, University of Bern,

Freiestrasse 3, 3012 Bern, Switzerland

**E-Mail: jean-louis.reymond@unibe.ch.*

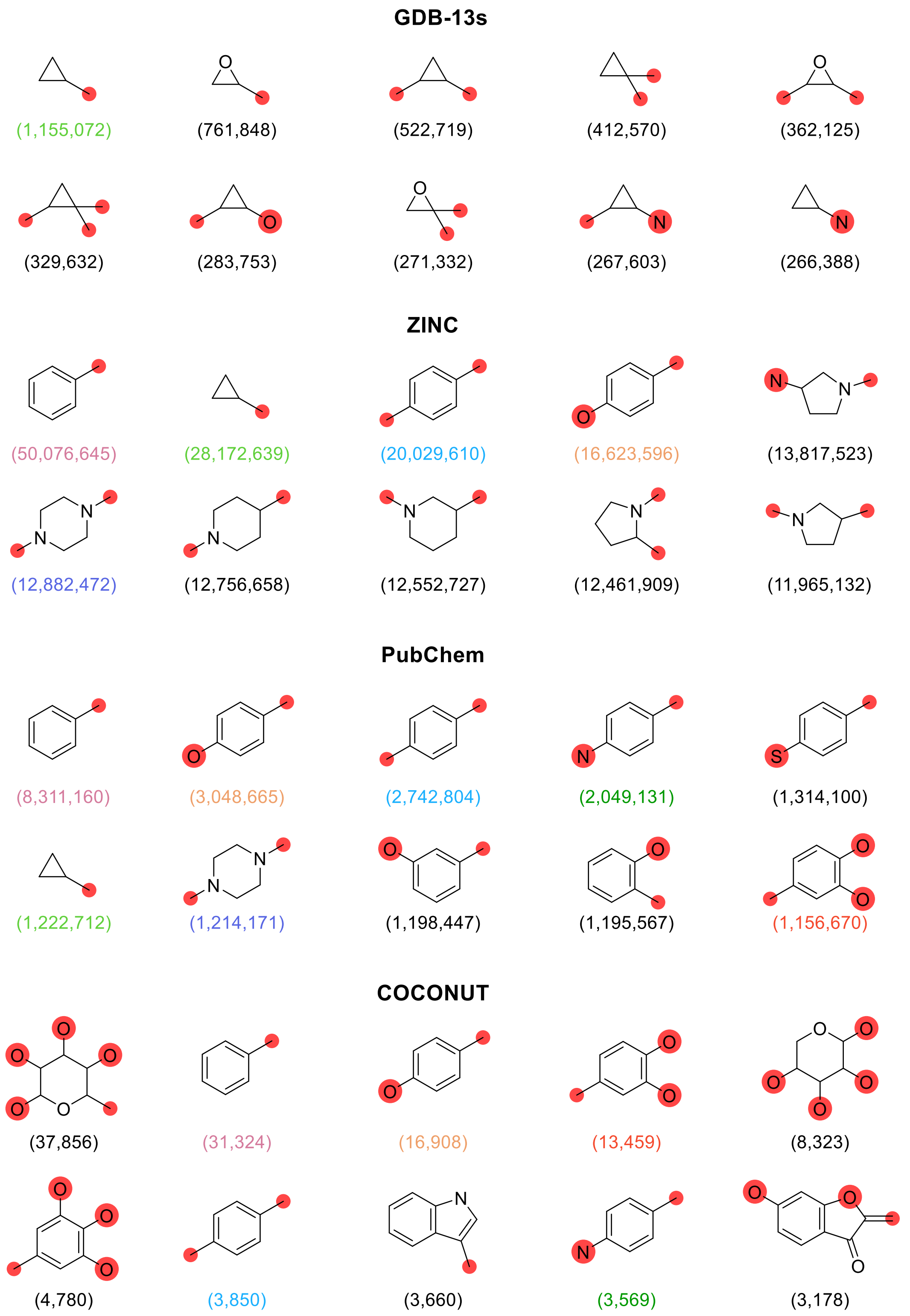

Figure S1. Top-10 most populated RFs in various databases. RFs are displayed by order of appearance in the frequency-sorted list across the four databases. A color-code has been added to the numbering of RFs appearing several times to facilitate comparison across different databases.

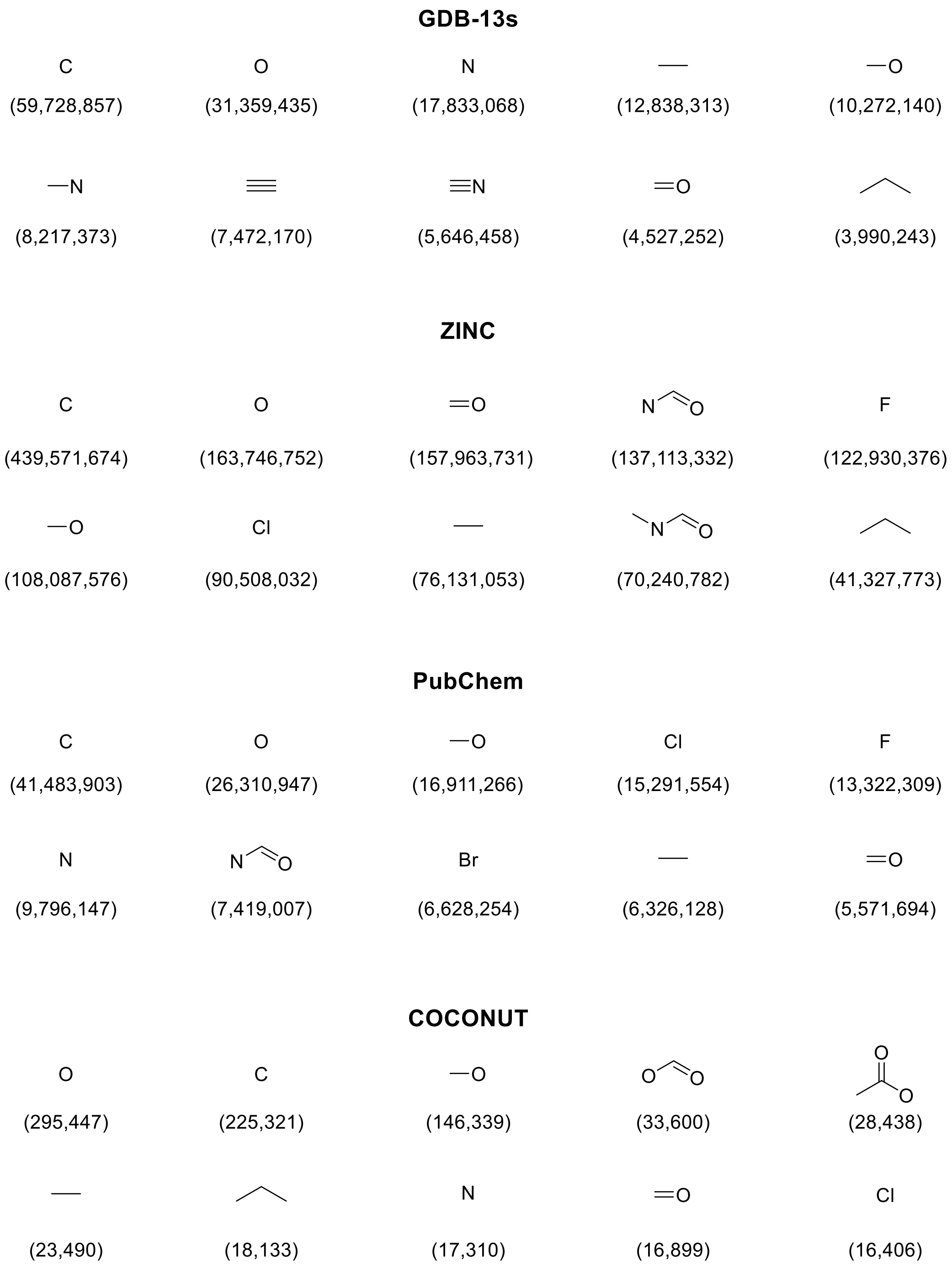

Figure S2. Top-10 most populated AFs in in various databases. AFs are displayed by order of appearance in the frequency-sorted list across the four databases.

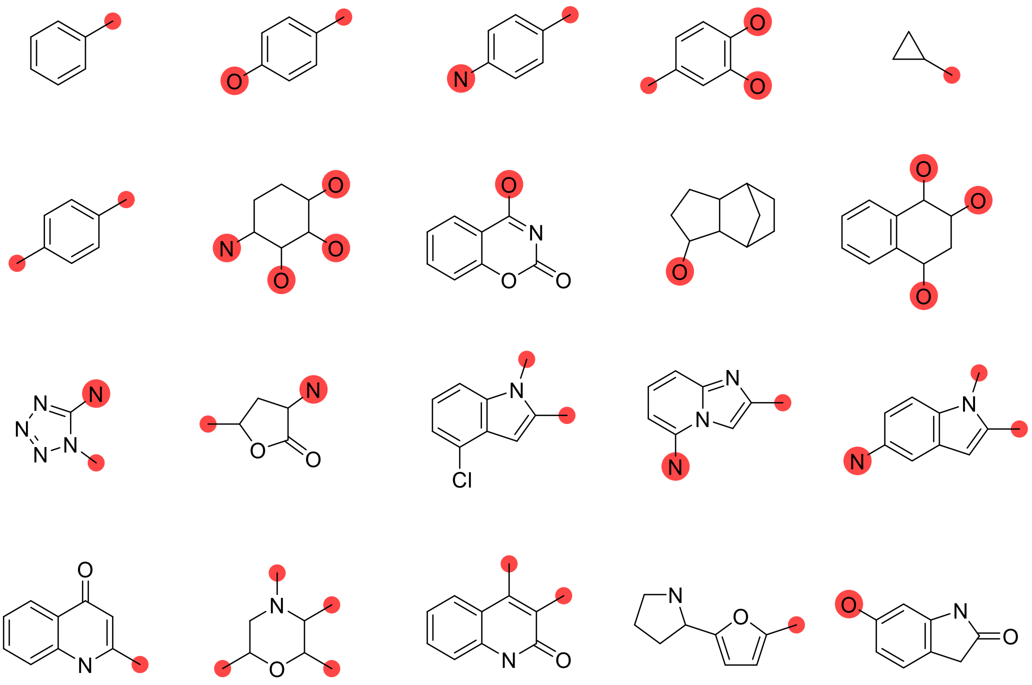

Figure S3. Top-20 most populated RFs shared by the different databases (GDB-13s, ZINC, PubChem, and COCONUT).

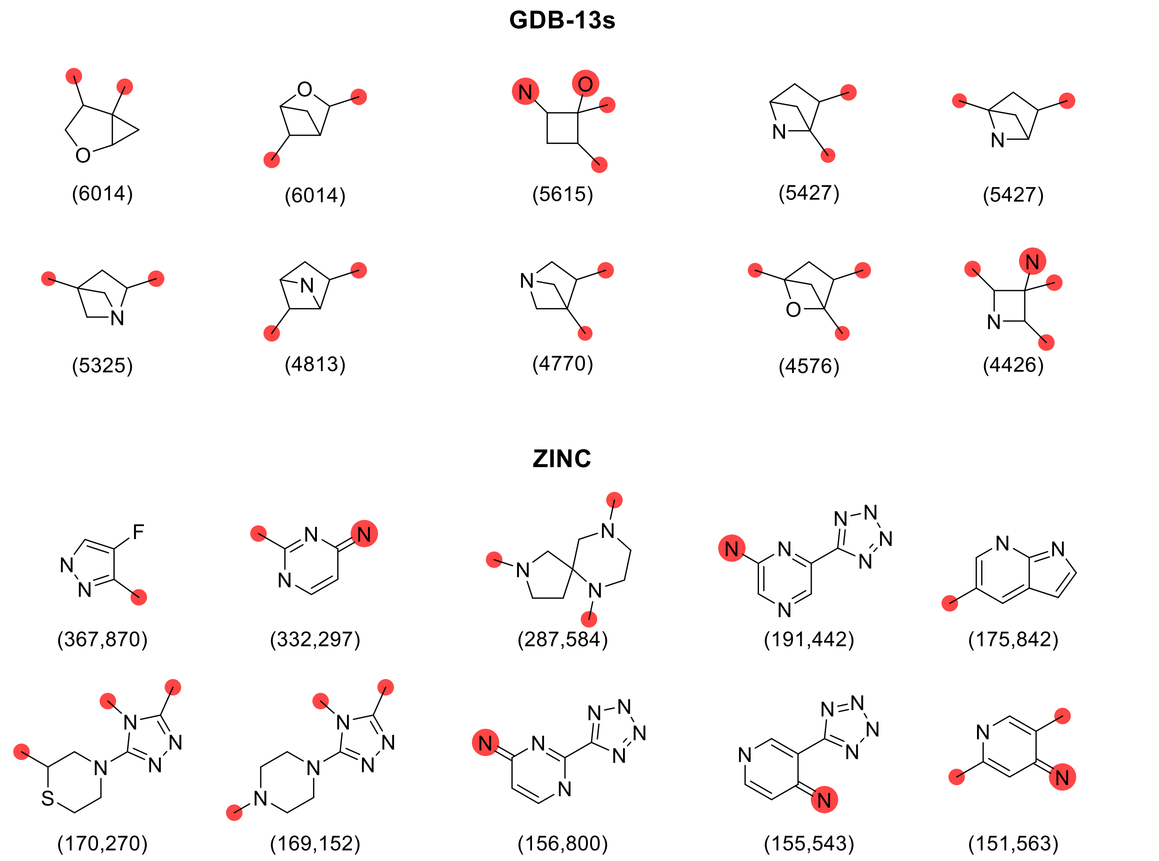

Figure S4. (**a**) Top-10 eRFs in GDB-13s and ZINC, occurrences of the obtained eRFs are indicated in parentheses.

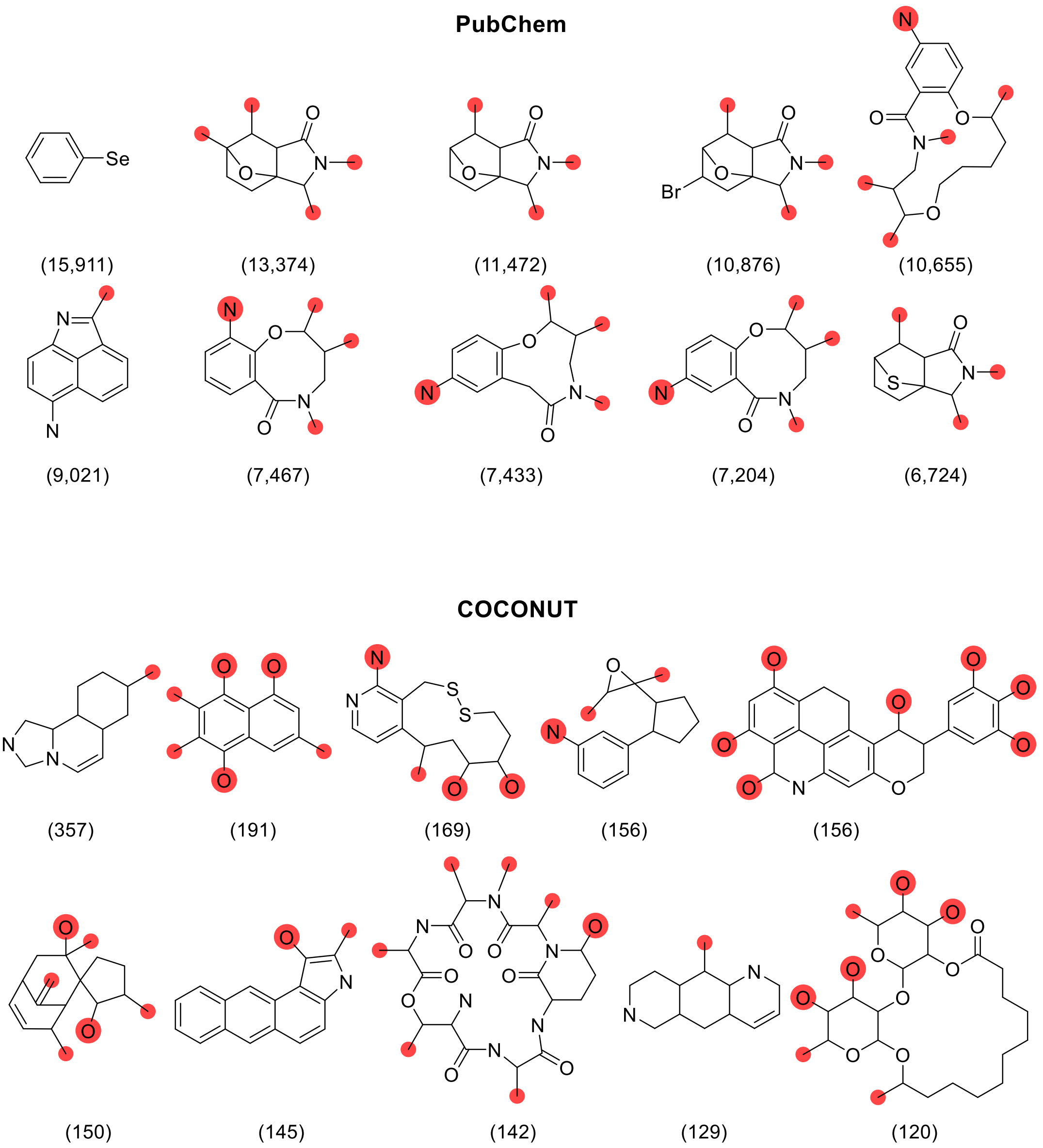

Figure S4. (**b**) Top-10 eRFs in PubChem and COCONUT, occurrences of the obtained eRFs are indicated in parentheses.

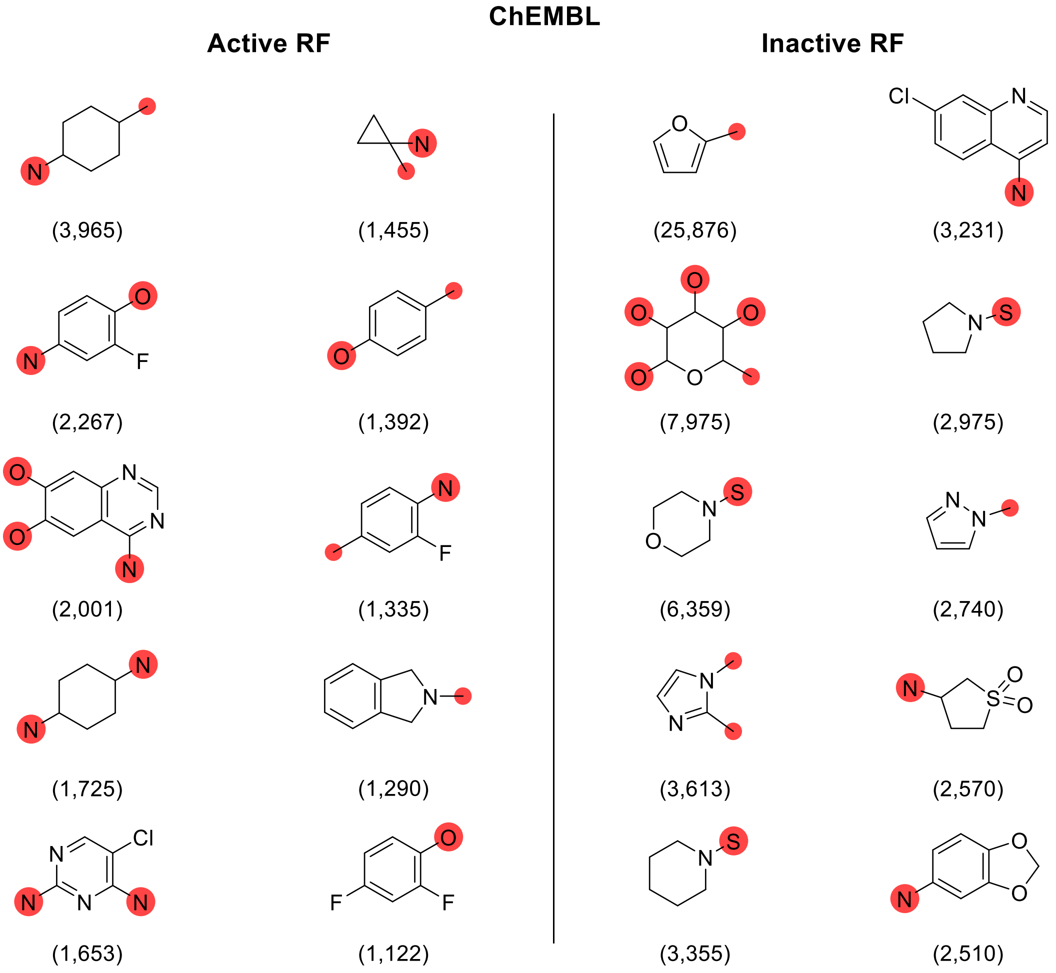

Figure S5. Top-10 most frequent RFs in the active ($R_{bioact} \geq 4$) and inactive ($R_{bioact} \leq 0.25$) ChEMBL subsets annotated with total occurrences of the RF in ChEMBL.

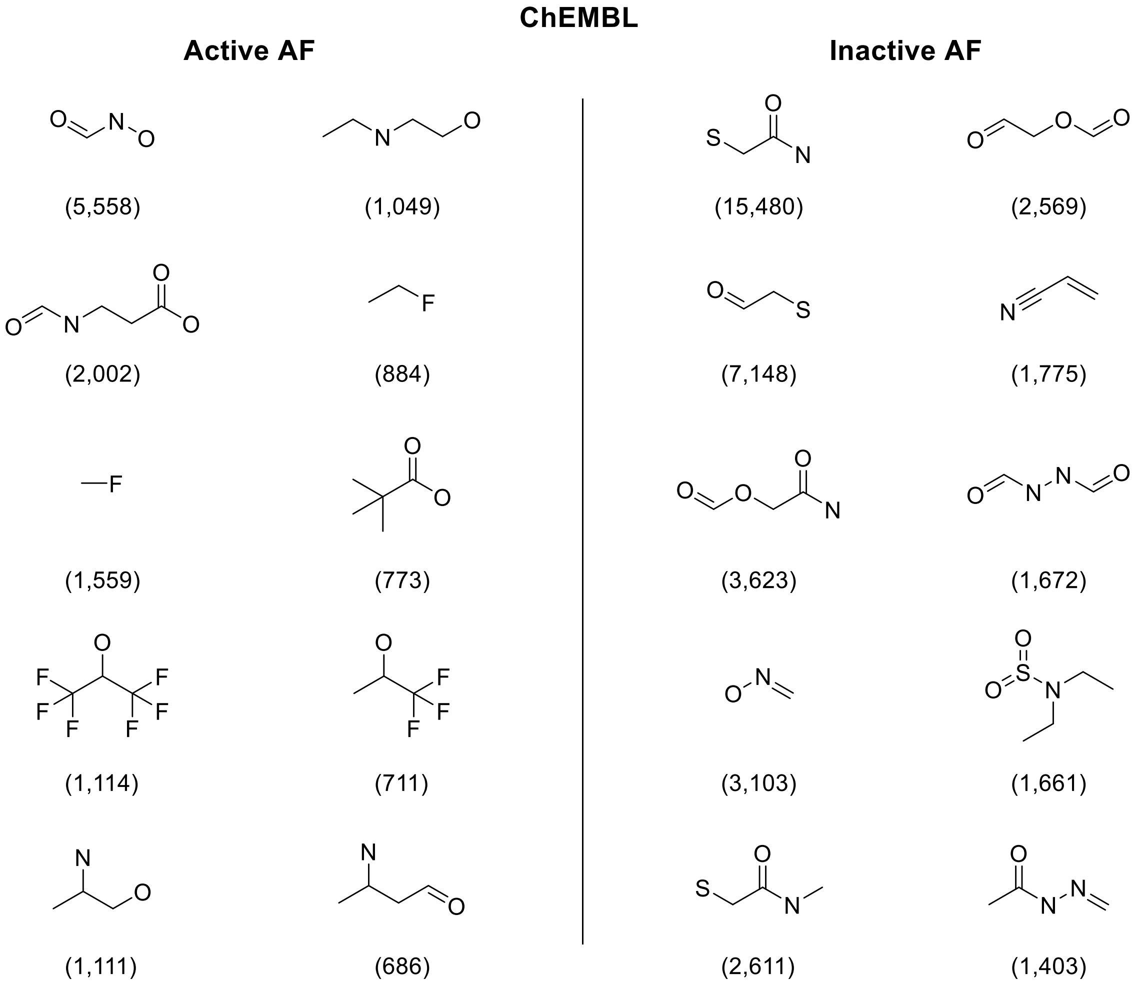

Figure S6. Top-10 most frequent AFs in the active ($R_{\mathrm{bioact}} \geq 4$) and inactive ($R_{\mathrm{bioact}} \leq 0.25$) ChEMBL subsets annotated with total occurrences of each AF in ChEMBL.

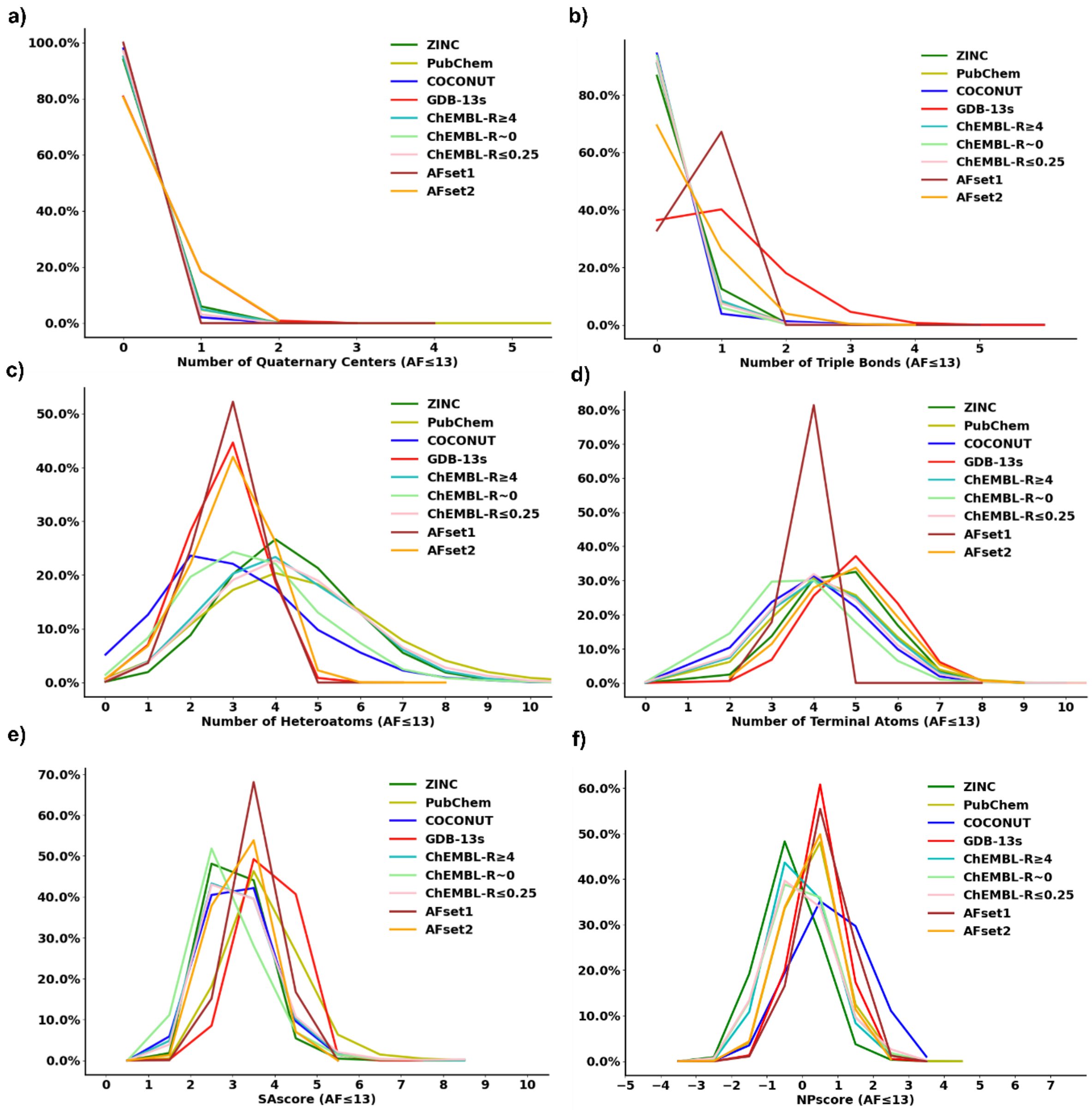

Figure S7. Frequency histograms of AFs from the various databases and subsets for (**a**) number of quaternary centers, (**b**) number of triple bonds, (**c**) number of heteroatoms, (**d**) number of terminal atoms, (**e**) SAscore, and (**f**) NPscore.

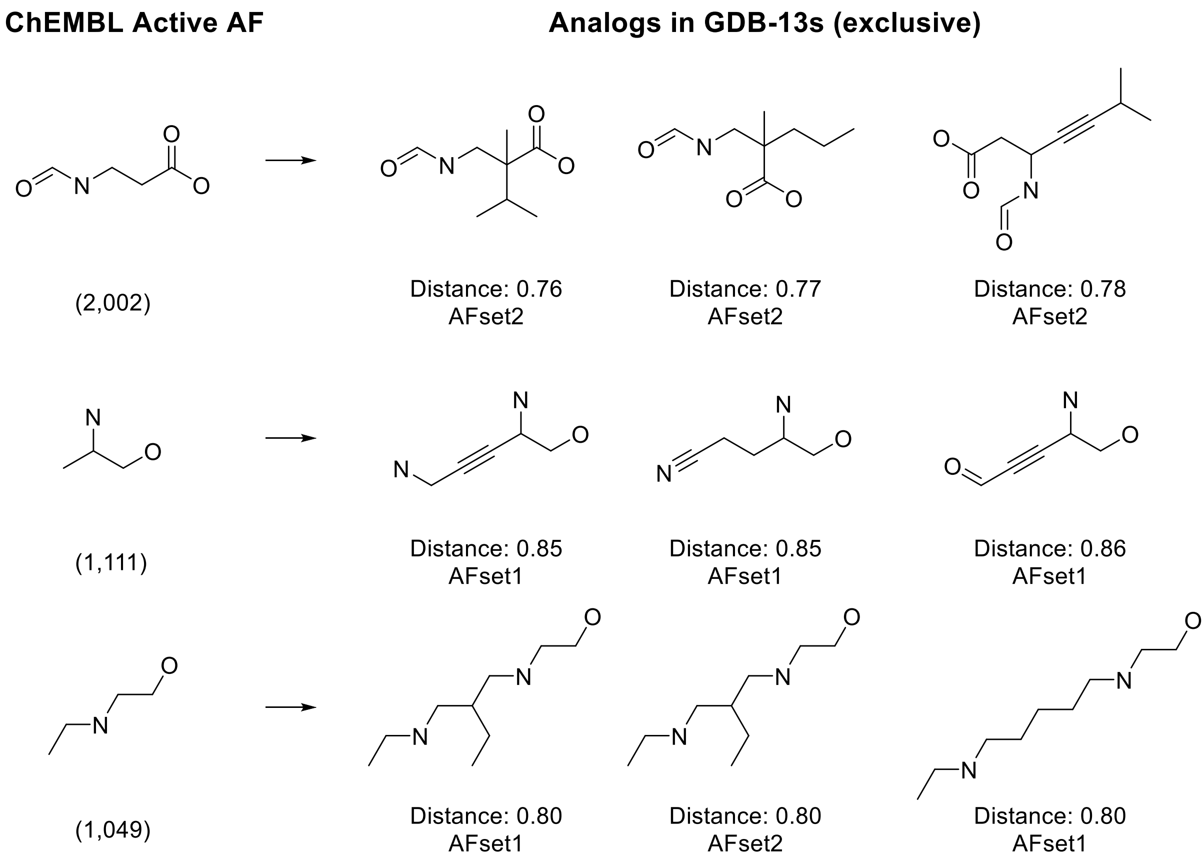

Figure S8. Analogs of highly active ChEMBL AFs found in the subsets of GDB-13s (**AFset1**/**AFset2**). Total occurrences of the ChEMBL AFs, or the distances between the analogs and the targets are indicated in parentheses.
